# ECONOMIC INSECURITY DURING THE COVID-19 PANDEMIC AMONG HEALTH CARE WORKERS BY EDUCATIONAL ATTAINMENT

**DOI:** 10.1093/geroni/igae098.3238

**Published:** 2024-12-31

**Authors:** Tracy Mroz, Ben Dunlap, Bianca Frogner

**Affiliations:** University of Washington, Seattle, Washington, United States; University of Washington, Seattle, Washington, United States; University of Washington, Seattle, Washington, United States

## Abstract

The COVID-19 pandemic had unprecedented impacts on healthcare workers. Prior to the pandemic, healthcare workers in lower skilled jobs (e.g., nursing assistants, home care aides) versus higher skilled jobs (e.g., physicians, nurses) were more likely to experience economic insecurity. We examined economic insecurity among healthcare workers between July 2021 and August 2023 via secondary analysis of Household Pulse Survey data. We used multivariable logistic regression to investigate the relationship between economic insecurity and educational attainment as a proxy for higher versus lower skilled workers, controlling for other sociodemographic characteristics. Of the 108,206 respondents who reported working in healthcare, representing a weighted sample of 10,656,917 healthcare workers, 49.5% had less than an associate degree, 10.0% had an associate degree, 21.7% had a bachelor’s degree, and 18.9% had a master’s degree or greater. Among workers with less than an associate degree, associate degree, bachelor’s degree, and master’s degree or higher: an estimated 78.7%, 69.9%, 56.1%, and 40.0% reported difficulty with usual expenses; 53.6%, 42.7%, 27.6%, and 17.4% reported food insufficiency; and 11.1%, 8.1%, 4.6%, and 2.8% reported being behind on their rent or mortgage payments, respectively. Lower educational attainment was significantly associated with greater economic insecurity across all measures. This relationship was attenuated, but still significant, after controlling for other sociodemographic characteristics. Lower skilled workers are critical to the healthcare workforce, especially in long-term care. Policies to promote economic security among these workers are needed to ensure a robust workforce is available to care for older adults.

